# Ginsenoside Rk1 alleviates lipopolysaccharide (LPS)-induced cognitive impairment by modulating synaptic plasticity

**DOI:** 10.3389/fphar.2025.1747574

**Published:** 2026-01-21

**Authors:** Xuesong Zhang, Funan Ning, Biqun Zhang, Jiaqi Ji, Junzuo Zheng, Xiaosong Hu, Zhiang Liu, Li Ding, Ping Wang, Zhou Lan

**Affiliations:** 1 School of Pharmacy, Hubei University of Chinese Medicine, Wuhan, China; 2 Department of Pharmacy, Hubei Provincial Hospital of Traditional Chinese Medicine, Hubei Province Academy of Traditional Chinese Medicine, Affiliated Hospital of Hubei University of Chinese Medicine, Wuhan, China; 3 Hubei Shizhen Laboratory, Hubei University of Chinese Medicine, Wuhan, China; 4 Engineering Research Center of TCM Protection Technology and New Product Development for the Elderly Brain Health, Ministry of Education, School of Basic Medicine, Hubei University of Chinese Medicine, Wuhan, China

**Keywords:** cognitive dysfunction, Ginsenoside Rk1, inflammation, network pharmacology, synaptic plasticity

## Abstract

**Background:**

This study aimed to comprehensively investigate the therapeutic effects of ginsenoside Rk1 on LPS-induced cognitive impairment and elucidate its underlying mechanisms, with a particular focus on synaptic plasticity and related signaling pathways, thereby providing robust theoretical and experimental support for its neuroprotective application.

**Research Methods:**

Network pharmacology identified potential therapeutic targets and pathways of ginsenoside Rk1 relevant to inflammation-induced cognitive impairment, and molecular docking assessed its binding affinity with key predicted proteins. *In vitro*, mouse bone marrow-derived macrophages (BMDMs) were used to determine the optimal non-cytotoxic concentration of ginsenoside Rk1 via CCK-8 assay. LPS and ATP were used to induce inflammation, and ELISA and RT-qPCR quantified pro-inflammatory cytokines and mRNA expression of Akt isoforms. For *in vivo* validation, male C57BL/6 mice were administered ginsenoside Rk1 (at an optimal dose of 20 mg/kg·d^−1^, i.g.) for 21 days, with LPS (500 μg/kg·d^−1^, i.p.) challenging on Day 22 and continued treatment for 7 days post-LPS. Cognitive function was assessed using the Morris water maze (MWM). Hippocampal samples were then analyzed for inflammatory factors, synaptic protein expression (PSD-95, SYN by RT-qPCR and immunofluorescence), microglial activation (Iba1 immunofluorescence), and dendritic spine density (Golgi staining).

**Results:**

Network pharmacology successfully identified significant overlaps between ginsenoside Rk1 targets and pathways associated with inflammation and cognitive impairment, prominently featuring the PI3K/Akt pathway. Molecular docking simulations confirmed strong binding affinities between ginsenoside Rk1 and key proteins in this pathway. *In vitro*, ginsenoside Rk1 significantly reduced LPS/ATP-induced levels of TNF-α, IL-1β, and IL-6, and attenuated the upregulation of Akt1, Akt2, and Akt3 mRNA expression. *In vivo*, ginsenoside Rk1 treatment profoundly improved spatial learning and memory in LPS-challenged mice. This cognitive improvement was paralleled by a significant attenuation of hippocampal neuroinflammation. Crucially, ginsenoside Rk1 significantly reversed LPS-induced synaptic dysfunction, characterized by increased mRNA and protein expression of PSD-95 and SYN, and a marked elevation in neuronal dendritic spine density in the hippocampus.

**Conclusion:**

This study provides compelling evidence that ginsenoside Rk1 effectively alleviates LPS-induced cognitive dysfunction by ameliorating neuroinflammation and significantly enhancing synaptic plasticity. The mechanistic insights suggest that these neuroprotective effects are mediated, at least in part, through the modulation of the PI3K/Akt signaling pathway.

## 1 Introduction

Inflammation is a fundamental pathological process intricately involved in numerous diseases and exerts a profound impact on cognitive function. Conditions such as viral encephalitis, bacterial meningitis, and systemic lupus erythematosus consistently demonstrate a strong association between inflammatory states and cognitive deficits, mimicking the systemic inflammatory environment simulated by lipopolysaccharide (LPS) models (Carroll et al., 2024; Garber et al., 2019; Lucas et al., 2016). Chronic or acute inflammation can culminate in cognitive impairment, severely diminishing patients’ quality of life. The etiology of this impairment is multifaceted, yet neuronal synapses-the critical junctions for information transmission-are central to cognitive processes such as learning and memory (Madar et al., 2025). Growing evidence underscores that inflammation detrimentally affects both the structure and function of synapses, establishing it as a pivotal driver in the progression of cognitive impairment (Jafari et al., 2021; Mandolesi et al., 2015; Patterson, 2015). Consequently, elucidating the mechanisms governing synapse loss under inflammatory conditions is paramount for developing effective therapeutic strategies.

Mechanistically, inflammation perturbs synaptic integrity through convergent pathways involving glial activation and cytokine release. In an inflammatory state, activated microglia release pro-inflammatory cytokines, prominently interleukin-1β (IL-1β) and tumor necrosis factor-α (TNF-α) (Sun et al., 2017). These cytokines exert direct adverse effects on synapses, manifesting as a reduction in dendritic spine density, which signifies decreased inter-neuronal connectivity (Heneka et al., 2015). Furthermore, inflammation compromises synaptic stability by disrupting the expression of key proteins such as postsynaptic density-95 (PSD-95) (Mir S et al., 2014). Given that PSD-95 is vital for anchoring glutamate receptors (El-Husseini et al., 2000), its downregulation inevitably leads to functional abnormalities in glutamatergic signaling (Béïque et al., 2006). Thus, targeting these inflammatory pathways to preserve synaptic plasticity represents a promising avenue for intervention.

The botanical drug ginseng has been utilized in traditional Chinese medicine (TCM) for centuries. Ginsenoside Rk1, a metabolite derived from ginseng via high-temperature processing, exhibits diverse pharmacological activities, including anti-apoptotic, anti-tumor, anti-insulin resistance, and antibacterial properties (Elshafay et al., 2017; Kwak and Pyo, 2016; Lee S, 2014; Ponnuraj et al., 2014; Xiong et al., 2021). Despite these established benefits, it remains elusive whether Ginsenoside Rk1 possesses the specific capacity to ameliorate inflammation-induced cognitive impairment. More importantly, it is unclear if this potential protective effect is mediated by the improvement of synaptic plasticity and the restoration of synaptic proteins like PSD-95.

Therefore, the present study employed a comprehensive network pharmacology approach combined with experimental validation to: (1) systematically screen for potential therapeutic targets of the metabolite Ginsenoside Rk1 associated with inflammation-induced cognitive impairment; (2) elucidate the underlying signaling pathways via Gene Ontology (GO) and KEGG enrichment analyses; and (3) validate these mechanisms. Specifically, molecular docking was utilized to predict binding affinities, *in vitro* experiments in bone marrow-derived macrophages (BMDMs) were conducted to verify regulatory effects, and *in vivo* animal assessments were performed to confirm the efficacy of Ginsenoside Rk1 in alleviating cognitive dysfunction. This study aims to provide a robust theoretical and experimental foundation for the clinical application of Ginsenoside Rk1.

## 2 Materials and methods

### 2.1 Network pharmacology analysis

The potential active targets of Ginsenoside Rk1 were predicted using a multi-database approach integrating the Comparative Toxicogenomics Database (CTD) (https://ctdbase.org/), PharmMapper (https://www.lilab-ecust.cn/pharmmapper/), and SwissADME (http://www.swissadme.ch/). Disease-related targets associated with “inflammation” and “cognitive impairment” were retrieved from the GeneCards (https://www.genecards.org/), PharmGKB (https://www.clinpgx.org/), and Online Mendelian Inheritance in Man (OMIM) (https://www.omim.org/). Common targets shared by inflammation and cognitive impairment were identified using Venny (https://bioinfogp.cnb.csic.es/tools/venny/index.html). Subsequently, the intersection of Ginsenoside Rk1 active targets and these common disease targets was determined. Overlapping targets were submitted to the STRING database (https://cn.string-db.org) (species: *Homo sapiens*; confidence score ≥0.4) to construct a protein-protein interaction (PPI) network, which was visualized using Cytoscape 3.9.1. Key targets were identified based on node degree and connectivity. Finally, Gene Ontology (GO) and Kyoto Encyclopedia of Genes and Genomes (KEGG) pathway enrichment analyses were performed using the DAVID 6.8 database (https://davidbioinformatics.nih.gov/). Visualization of the top 10 GO terms and top 20 KEGG pathways was generated using an online bioinformatics platform (https://www.bioinformatics.com.cn/).

### 2.2 Molecular docking

Molecular docking, a pivotal computational technique in modern drug discovery (Kitchen et al., 2004), was performed to evaluate the binding affinity between Ginsenoside Rk1 (ligand) and key disease target proteins. Simulations were conducted using AutoDock Vina 1.1.2. Prior to simulation, the three-dimensional structures of the Ginsenoside Rk1 ligand and selected target proteins were prepared and converted to PDBQT format utilizing PyRx. Docking boxes were defined to encompass the putative active sites of each target protein. A binding energy value below −6.0 kcal/mol was considered indicative of a strong and favorable ligand-protein affinity (Li et al., 2023).

### 2.3 Cell culture

Mouse bone marrow-derived macrophages (BMDMs) were isolated from the femurs and tibias of 8–10-week-old male C57BL/6J mice. BMDMs were cultured in high-glucose Dulbecco’s Modified Eagle’s Medium (DMEM) supplemented with 10% fetal bovine serum (FBS, Gibco), 100 U/mL penicillin (Dalian Meilun Biotechnology Co., Ltd., Dalian, China), 100 μg/mL streptomycin (Dalian Meilun Biotechnology Co., Ltd.), and 20 ng/mL macrophage colony-stimulating factor (M-CSF, ABclonal Biotechnology Co., Ltd., Wuhan, China). For enzyme-linked immunosorbent assay (ELISA) experiments, BMDMs were seeded into 96-well plates at a density of 1.5 × 10^6^ cells/mL, with 200 μL of cell suspension per well. For reverse transcription-quantitative polymerase chain reaction (RT-qPCR) experiments, BMDMs were seeded into 6-well plates at a density of 4 × 10^6^ cells/mL, with 2 mL of cell suspension per well (equivalent to 8 × 10^6^ cells per well). Cultures were maintained at 37 °C in a humidified 5% CO_2_ atmosphere. Half of the medium was replaced on day 3, and a complete medium change was performed on day 5. Experiments were conducted on day 7 to ensure optimal differentiation.

### 2.4 Cell viability assay

BMDMs were seeded into 96-well plates (5 × 10^4^ cells/well) and cultured for 7 days. Cells were then treated with various concentrations of Ginsenoside Rk1 for 24 h (100 μL per well). Cell viability was assessed using the Cell Counting Kit-8 (CCK-8; Beijing Solarbio Science & Technology Co., Ltd.). After adding 10 μL of CCK-8 reagent, plates were incubated for 4 h at 37 °C. Absorbance was measured at 450 nm using a microplate reader. Viability was calculated as a percentage relative to the untreated control group (set as 100%).

### 2.5 *In vitro* modeling and treatment

BMDMs were divided into three groups: control, model, and Ginsenoside Rk1. Cells in the Ginsenoside Rk1 group were pretreated with Ginsenoside Rk1 (40 μg/mL) for 3 h. Following pretreatment, both the model and Ginsenoside Rk1 groups were stimulated with LPS (100 ng/mL) for 3 h to induce inflammation. The control group received serum-reduced medium. Subsequently, the model group was treated with adenosine triphosphate (ATP, 5 μmol/mL, Sigma-Aldrich) to activate the inflammsome, while the Ginsenoside Rk1 group received combined treatment (40 μg/mL Ginsenoside Rk1 + 5 μmol/mL ATP). Supernatants were collected 3 h post-ATP treatment for ELISA (TNF-α, interleukin-6), and cells were harvested for reverse transcription-quantitative polymerase chain reaction (RT-qPCR) assays.

### 2.6 Animals

Thirty-six 8-week-old male C57BL/6 mice were obtained from Liaoning Changsheng Biotechnology Co., Ltd. Mice were randomly divided into three groups (n = 12 mice/group): Control, Model, and Ginsenoside Rk1. Ginsenoside Rk1 was purchased from Wuhan Tianzhi Biotechnology Co., Ltd. All procedures were approved by the Animal Care and Use Committee of Hubei University of Chinese Medicine (Ethics Approval Number: HUCMS21088478), fully complying with the guidelines outlined in the National Institutes of Health Guide for the Care and Use of Laboratory Animals. Mice were housed under standard conditions (23 °C ± 2 °C; 12-h light/dark cycle) with *ad libitum* access to food and water. Animals were habituated for 1 week prior to experiments. A schematic of the experimental design is shown in Figure 3A.

### 2.7 Lipopolysaccharide (LPS) challenge experiment

Mice in the control and model groups received daily intragastric gavage (i.g.) of normal saline. The Ginsenoside Rk1 group received daily i.g. administration of Ginsenoside Rk1 (dissolved in normal saline) at a dose of 20 mg/kg body weight (BW). This regimen continued for 21 days. On day 22, the model group was subjected to an inflammatory insult via a single intraperitoneal (i.p.) injection of LPS (500 μg/kg BW, Sigma Aldrich, St. Louis, MO, United States). The control group received an equivalent volume of saline i.p. In the Ginsenoside Rk1 group, drug administration was performed 3 h prior to the LPS challenge and continued daily until day 7 post-LPS administration. Tissue samples were collected 6 h following the final procedure.

### 2.8 Behavioral tests: Morris water maze (MWM)

The MWM test was performed to assess spatial learning and memory using a specialized tracking system (Model XR-XM101, Shanghai XinRuan Information Technology Co., Ltd.). The test was performed as previously described (Lan et al., 2012). The test comprised 5 days of training trials followed by a probe trial on day 6. The apparatus consisted of a circular pool (100 cm diameter) with a hidden platform. Mice were released from different quadrants, and the escape latency was recorded (capped at 60 s). On day 6, the platform was removed, and the time spent in the target quadrant and the number of platform crossings were recorded to evaluate memory consolidation. Swimming speed was monitored to exclude motor deficits.

### 2.9 RT-qPCR

Total RNA was isolated from cell or tissue samples using TRIzol reagent (Vazyme Biotech Co., Ltd.) according to the manufacturer’s instructions. Isolated RNA was subsequently resuspended in diethyl pyrocarbonate (DEPC)-treated, ribonuclease (RNase)-free water (Vazyme). Specific primer sequences for PCR amplification are listed in Table 1. Complementary DNA (cDNA) synthesis was performed using HiScript III RT SuperMix for qPCR (Vazyme), which includes gDNA wiper to ensure removal of genomic DNA contamination, catalyzing reverse transcription from RNA templates. qPCR was then conducted using Taq Pro Universal SYBR qPCR Master Mix (Vazyme). Relative quantification of gene expression was determined using the comparative cycle threshold 2^(−ΔΔCT)^ method. The calculation formula was applied as follows:

- ΔCT = CT (a target gene) − CT (a reference gene);
- ΔΔCT = ΔCT (a target sample) − ΔCT (a reference sample);
- Relative RNA abundance = 2^(−ΔΔCT)^.

**TABLE 1:** Primer sequences used for RT-qPCR analysis.

Gene	Forward primer (5′–3′)	Reverse primer (5′–3′)
GAPDH	CCT​CGT​CCC​GTA​GAC​AAA​ATG	TGA​GGT​CAA​TGA​AGG​GGT​CGT
IL-1β	GCA​TCC​AGC​TTC​AAA​TCT​CGC	GCA​TCC​AGC​TTC​AAA​TCT​CGC
TNF-α	GTC​TAC​TGA​ACT​TCG​GGG​TGA​TC	TCC​TCC​ACT​TGG​TGG​TTT​GTG​A
IL-6	GAC​AAA​GCC​AGA​GTC​CTT​CAG​AGA​G	CTA​GGT​TTG​CCG​AGT​AGA​TCT​C
PSD-95	GCA​GGT​TGC​AGA​TCG​GAG​AC	ACTGATCTCATTGTCCAGGTGCTF
SYN	ACC​TCG​GTG​GTG​TTT​GGC​TT	TGCCCGTAATCGGGTTGA
Akt1	GCT​CTT​CTT​CCA​CCT​GTC​TCG	CGC​AGA​ATG​TCT​TCA​TAG​TGG​C
Akt2	GGC​AAG​GTC​ATT​CTG​GTT​CGA	GCA​TAG​GCG​GTC​ATG​GGT​CT
Akt3	TGA​GGA​CCG​CAC​ACG​TTT​CTA​T	GCA​AAG​CCC​AAA​ATC​CGT​AA

This table lists the forward and reverse primer sequences (5′–3′) for the seven genes analyzed in this study (GAPDH, IL-1β, TNF-α, IL-6, PSD-95, SYN, Akt1, Akt2, Akt3).

### 2.10 Immunofluorescence staining

Brain tissues were fixed in 4% paraformaldehyde, embedded in paraffin, and sectioned (4 µm). Sections were incubated overnight at 4 °C with primary antibodies against Iba1 (GB113502, 1:5000, Servicebio, Wuhan, China) and PSD-95(GB11277, 1:2000, Servicebio), followed by incubation with a Cy3 conjugated Goat Anti-Rabbit IgG (H + L) (1:300, GB21303, Servicebio) for 50 min at room temperature. Nuclei were counterstained with DAPI. Images were acquired using a fluorescence microscope (NIKON ECLIPSE C1 with NIKON DSU3, Nikon Corporation, Tokyo, Japan) and analyzed using ImageJ software.

### 2.11 Golgi staining

Golgi staining, a classic and invaluable technique for visualizing neuronal morphology, particularly dendritic and dendritic spine characteristics, is widely utilized in neurodegenerative disorder research (Liu et al., 2014). Brains from mice (n = 3 per group) were processed using a commercial Golgi-staining kit (Wuhan Servicebio Technology Co., Ltd., Wuhan, China) strictly according to the manufacturer’s instructions. Following impregnation and staining, Golgi-stained brains were coronally sectioned at a thickness of 60 μm using a vibratome (LEICA VT 1000S) at room temperature. Sections were visualized and imaged under an upright microscope (3DHISTECH, Hungary) by two independent researchers who were rigorously blinded to the experimental group allocation. For quantitative dendritic spine analysis, segments of 20–30 μm from three tertiary dendrites, meticulously selected from the hippocampal region, were utilized. Specifically, three neurons per brain slice and three brain slices per animal were randomly chosen for robust quantitative analysis of dendritic spine density (Chunchai et al., 2016).

### 2.12 Measurement of pro-inflammatory cytokines

Pro-inflammatory cytokines, including TNF-α, IL-6 and IL-1β, in cell culture supernatants were measured using mouse ELISA kits (R&D Systems, Minneapolis, MN, United States) following the manufacturer’s instructions. Similarly, the levels of these pro-inflammatory cytokines in mouse hippocampus were detected using mouse ELISA kits (ABclonal Biotech Co., Ltd., China), also performed strictly according to the manufacturer’s protocols.

### 2.13 Statistical analysis

All data are presented as mean ± S.E.M. MWM escape latency data were analyzed using two-way ANOVA followed by Dunnett’s multiple comparisons test. Other data were analyzed using one-way ANOVA followed by Dunnett’s test. Analyses were performed using GraphPad Prism 10.0.3. A p-value <0.05 was considered statistically significant.

## 3 Result

### 3.1 Network pharmacology identifies the PI3K/Akt pathway as a key target for ginsenoside Rk1 in alleviating LPS-induced cognitive impairment

**FIGURE 1:**
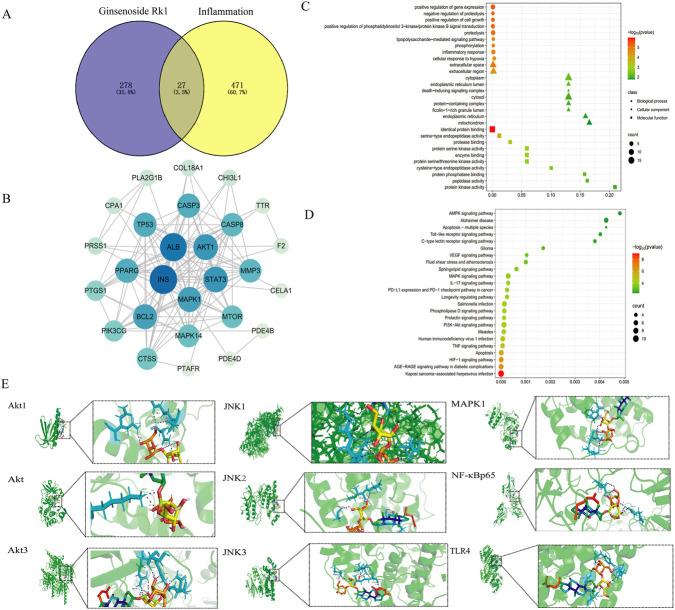
Results of network pharmacology analysis. **(A)** Venn diagram illustrating the intersection of active targets of Ginsenoside Rk1 and disease-related targets associated with inflammation and cognitive impairment. **(B)** Protein-protein interaction (PPI) network of 27 identified common targets, constructed and visualized using Cytoscape. **(C)** Top 10 enriched GO biological functions, highlighting the key biological processes modulated by the common targets. **(D)** Top 20 enriched KEGG pathways, indicating the most significantly affected signaling pathways. **(E)** Representative molecular docking visualizations of Ginsenoside Rk1 with selected target proteins, illustrating predicted binding poses and interactions.

A comprehensive network pharmacology approach identified 278 potential active targets of ginsenoside Rk1 and 471 targets associated with inflammation-induced cognitive impairment (Figure 1A). Venny analysis pinpointed 27 intersecting targets shared between the drug and the disease. These targets were mapped using the STRING database to construct a protein-protein interaction (PPI) network (Figure 1B). Subsequent Gene Ontology (GO) and Kyoto Encyclopedia of Genes and Genomes (KEGG) pathway analyses were performed using the DAVID 6.8 database; the top 10 GO terms and top 20 KEGG pathways are presented in Figures 1C,D, respectively.

Based on enrichment significance (p < 0.05), inflammation-related pathways—specifically TNF, NF-κB, PI3K/Akt, and MAPK—were selected for molecular docking. The docking simulations (Figure 1E; Table 2) revealed that proteins within the PI3K/Akt pathway exhibited the lowest binding energies (indicating high affinity) with ginsenoside Rk1. These findings suggest that ginsenoside Rk1 may alleviate LPS-induced cognitive impairment by modulating the PI3K/Akt signaling pathway.

**TABLE 2:** Molecular docking results of Ginsenoside Rk1 with core targets of NF-κB, MAPK, and PI3K/Akt Pathways.

Compound	Target protein	Binding energy (kcal‧mol^−1^)
Ginsenoside Rk1	TLR4	−7.4
	NF-κBp65	−8.2
	AKT1	−6.8
	AKT2	−9.5
	AKT3	−9.6
	JNK1	−8.5
	JNK2	−8.7
	JNK3	−7.9
	MAPK1	−8.9

This table summarizes the predicted binding energies (kcal·mol^−1^) of Ginsenoside Rk1 with selected key target proteins from the NF-κB, MAPK, and PI3K/Akt signaling pathways, indicating their binding affinity.

### 3.2 *In vitro* verification of ginsenoside Rk1 anti-inflammatory activity

**FIGURE 2:**
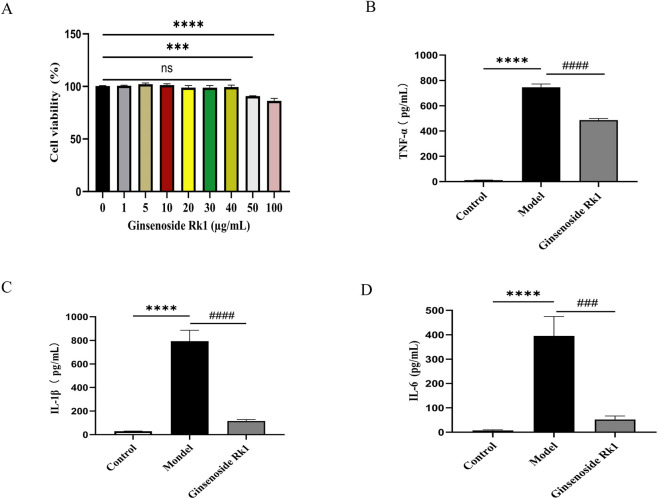
*In vitro* anti-inflammatory effects of Ginsenoside Rk1. **(A)** Cell viability of BMDMs after 24 h treatment with different concentrations of Ginsenoside Rk1. Data are presented as mean ± S.E.M. (n = 6). Statistical significance: ***P < 0.001 and ****P < 0.0001 vs. control group (0 μg/mL Ginsenoside RK1). **(B–D)** Levels of pro-inflammatory cytokines in culture supernatants. **(B,D)** For TNF-α and IL-6 analysis, cells were pretreated with ginsenoside Rk1 (40 μg/mL) followed by LPS stimulation (100 ng/mL). **(C)** For IL-1β analysis, cells were pretreated with ginsenoside Rk1 (40 μg/mL), stimulated with LPS (100 ng/mL), and subsequently activated with ATP (5 μmol/mL). Data are presented as mean ± S.E.M. (n = 6). Statistical significance: ***P < 0.001, ****P < 0.0001 vs. Control group; ####P < 0.0001 vs. Model group. Statistical analyses were performed using one-way ANOVA followed by Dunnett’s test.

The anti-inflammatory efficacy of Ginsenoside Rk1 was validated in BMDMs. A concentration of 40 μg/mL was selected for subsequent experiments based on cell viability assays, which showed no significant cytotoxicity at this dose (Figure 2A).

Compared to the control group, the model group exhibited significantly elevated levels of pro-inflammatory cytokines, including TNF-α (Figure 2B), IL-1β (Figure 2C), and IL-6 (Figure 2D). Pretreatment with Ginsenoside Rk1 significantly attenuated those inflammatory responses, reducing the secretion of TNF-α, IL-6, and IL-1β to levels compared to the model group. These results confirm the potent anti-inflammatory activity of ginsenoside Rk1 *in vitro*.

### 3.3 Ginsenoside Rk1 ameliorates LPS-induced cognitive impairment *in vivo*

**FIGURE 3:**
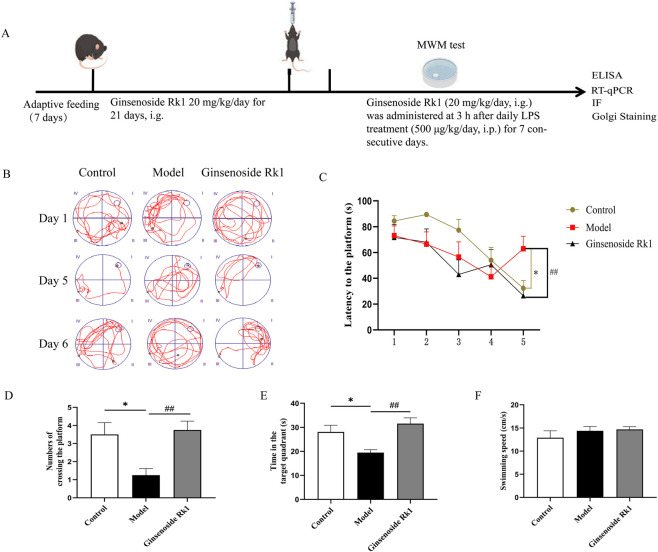
Ginsenoside Rk1 alleviates LPS-induced cognitive impairment *in vivo*. **(A)** Schematic diagram of the experimental design and timeline. **(B)** Representative swimming trajectories of mice during the MWM test on Day 1 (initial training), Day 5 (final training), and Day 6 (probe trial). **(C)** Escape latency during the 5-day training phase, reflecting spatial learning acquisition. **(D)** Number of platform crossings during the probe trial (Day 6), indicating spatial memory retrieval. **(E)** Time spent in the target quadrant during the probe trial (Day 6). **(F)** Average swimming speed, analyzed to rule out motor deficits. Data are presented as mean ± S.E.M. (n = 8). Statistical significance: *P < 0.05 vs. Control group; ^##^P < 0.01 vs. Model group. Statistical analyses were performed using two-way ANOVA (escape latency) or one-way ANOVA (other parameters) followed by Dunnett’s test.

MWM test was assessed to evaluate the effect of Ginsenoside Rk1 on spatial learning and memory. Representative swimming trajectories are shown in Figure 3B. During the training phase (Figure 3C), the model group displayed significantly prolonged escape latencies compared to the control group, indicating learning deficits. In contrast, Ginsenoside Rk1 treatment significantly reduced escape latency. In the probe trial on Day 6 (Figures 3D,E), the model group showed a marked reduction in the number of platform crossings and time spent in the target quadrant, indicative of impaired memory consolidation. Treatment with Ginsenoside Rk1 significantly reversed these deficits, increasing both platform crossings and time in the target quadrant. No significant differences in swimming speed were observed among the groups (Figure 3F), ruling out motor deficits as a confounding factor. These data indicate that Ginsenoside Rk1 effectively alleviates LPS-induced cognitive impairment.

### 3.4 Ginsenoside Rk1 mitigates LPS-induced neuroinflammation *in vivo*

**FIGURE 4:**
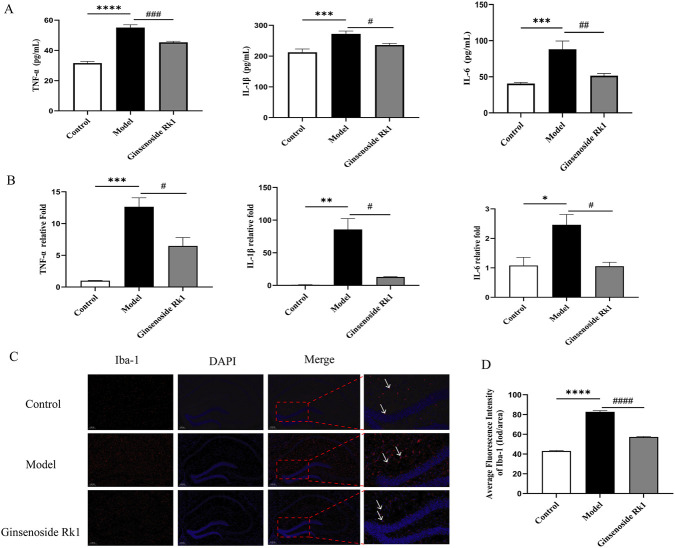
Ginsenoside Rk1 mitigates LPS-induced neuroinflammation in the hippocampus. **(A)** Protein levels of pro-inflammatory cytokines (TNF-α, IL-1β, and IL-6) in hippocampal tissue, measured by ELISA (n = 6). **(B)** Relative mRNA expression levels of pro-inflammatory cytokines in hippocampal tissue, analyzed by RT-qPCR (n = 3). **(C)** Representative immunofluorescence images of Iba1 (microglial marker) in the hippocampus. Scale bar: 200 μm. **(D)** Quantification of Iba1 immunofluorescence intensity. Data are presented as mean ± S.E.M. (n = 3 for **(B–D)**. Statistical significance: ^*^P < 0.05, ^**^P < 0.01, ^***^P < 0.001, ^****^P < 0.0001 vs. Control group; ^#^P < 0.05, ^###^P < 0.001, ^####^P < 0.0001 vs. Model group. Statistical analyses were performed using one-way ANOVA followed by Dunnett’s test.

To assess neuroinflammation, pro-inflammatory markers in the mouse hippocampus were quantified. ELISA results (Figure 4A) showed that LPS challenge significantly increased hippocampal levels of TNF-α, IL-6, and IL-1β compared to controls. Ginsenoside Rk1 treatment significantly suppressed these cytokines. Consistent with ELISA findings, RT-qPCR analysis (Figure 4B) demonstrated that Ginsenoside Rk1 significantly downregulated the mRNA expression of these pro-inflammatory factors in the hippocampus. Furthermore, immunofluorescence staining for Iba1 (Figure 4C) revealed increased microglial activation in the model group, which was notably attenuated by Ginsenoside Rk1. Collectively, these results demonstrate that Ginsenoside Rk1 inhibits LPS-induced neuroinflammation in the hippocampus.

### 3.5 Ginsenoside Rk1 ameliorates LPS-induced synaptic dysfunction

**FIGURE 5:**
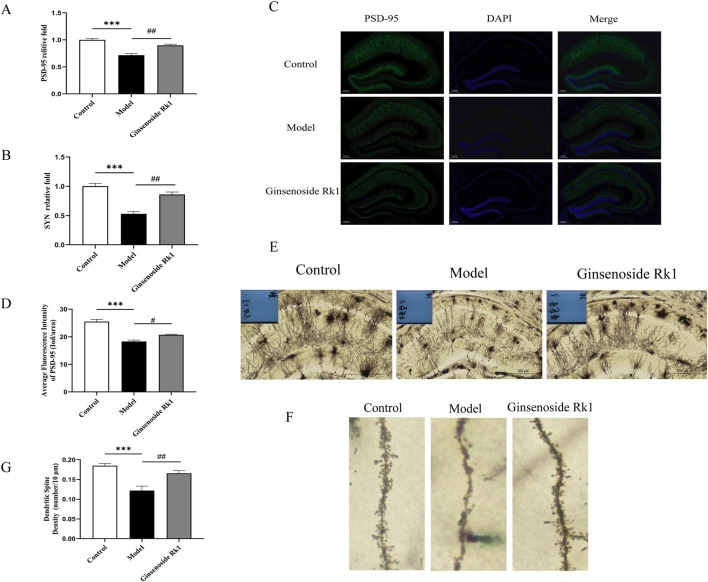
Ginsenoside Rk1 ameliorates LPS-induced synaptic dysfunction. **(A,B)** Relative mRNA expression levels of PSD-95 **(A)** and SYN **(B)** in hippocampal tissue (n = 3). **(C)** Representative immunofluorescence images of PSD-95 in the hippocampus. Scale bar: 200 μm. **(D)** Quantification of PSD-95 immunofluorescence intensity. **(E)** Representative Golgi staining images showing neuronal morphology in the hippocampus. Scale bar: 500 μm. **(F)** High-magnification images showing dendritic spines. Scale bar: 20 μm. **(G)** Quantification of dendritic spine density. Data are presented as mean ± S.E.M. (n = 3). Statistical significance: ^**^P < 0.01, ^***^P < 0.001 vs. Control group; ^#^P < 0.05, ^##^P < 0.01 vs. Model group. Statistical analyses were performed using one-way ANOVA followed by Dunnett’s test.

We further investigated the effect of Ginsenoside Rk1 on synaptic integrity. RT-qPCR analysis (Figures 5A,B) revealed significantly decreased mRNA expression of the synaptic proteins PSD-95 and synaptophysin (SYN) in the model group. Ginsenoside Rk1 treatment restored the expression of both genes to levels approaching those of the control group. Immunofluorescence staining corroborated these results, showing a significant reduction in PSD-95 density in the model group that was reversed by Ginsenoside Rk1 (Figures 5C,D).

Additionally, Golgi staining was employed to assess dendritic spine density in the hippocampus (Figures 5E–G). The model group exhibited a significant loss of dendritic spines compared to controls. However, Ginsenoside Rk1 treatment significantly preserved dendritic spine density. These findings indicate that Ginsenoside Rk1 protects against LPS-induced synaptic dysfunction and structural plasticity deficits.

### 3.6 Ginsenoside Rk1 alleviates LPS-induced cognitive impairment through modulation of the PI3K/Akt pathway

Network pharmacology analysis (Section 3.1) predicted the PI3K/Akt pathway as a primary mechanism of Ginsenoside Rk1. To validate this, the expression of key Akt isoforms was examined.

**FIGURE 6:**
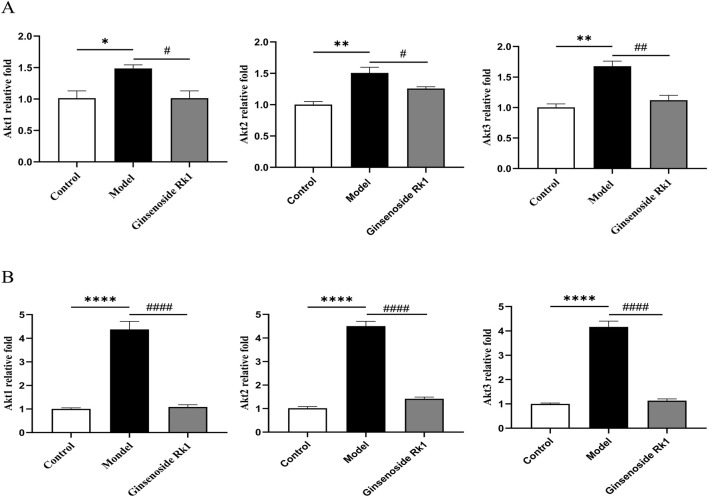
Ginsenoside Rk1 regulates the PI3K/Akt pathway. **(A)** Relative mRNA expression levels of Akt1, Akt2, and Akt3 in BMDMs (n = 6). **(B)** Relative mRNA expression levels of Akt1, Akt2, and Akt3 in hippocampal tissue (n = 6). Data are presented as mean ± S.E.M. (n = 6). Statistical significance: ^*^P < 0.05, ^**^P < 0.01, ^****^P < 0.0001 vs. Control group; ^#^P < 0.05, ^##^P < 0.01, ^####^P < 0.0001 vs. Model group. Statistical analyses were performed using one-way ANOVA followed by Dunnett’s test.

In BMDMs, RT-qPCR analysis (Figure 6A) showed that LPS stimulation significantly upregulated the mRNA expression of Akt1, Akt2, and Akt3. Ginsenoside Rk1 treatment significantly reversed this upregulation. Similarly, *in vivo* analysis of hippocampal tissue (Figure 6B) demonstrated that the LPS-induced elevation of Akt1, Akt2, and Akt3 mRNA levels was significantly inhibited by Ginsenoside Rk1. These consistent *in vitro* and *in vivo* findings support the hypothesis that Ginsenoside Rk1 alleviates cognitive impairment and neuroinflammation via modulation of the PI3K/Akt signaling pathway.

## 4 Discussion

Neuroinflammation is a pervasive pathological hallmark across a spectrum of neurological disorders, including Alzheimer’s disease, Parkinson’s disease, depression, and insomnia (Boyd et al., 2022; Gorji, 2022; Xia et al., 2021). In this context, our study investigated the therapeutic potential and underlying mechanisms of Ginsenoside Rk1 in a mouse model of LPS-induced cognitive dysfunction, which serves as a clinically relevant paradigm for systemic inflammation-related neurological impairment. Our findings demonstrate that Ginsenoside Rk1 significantly ameliorates synaptic plasticity impairments and cognitive deficits, primarily by modulating the PI3K/Akt signaling pathway.

We employed a comprehensive network pharmacology approach to elucidate the molecular targets of Ginsenoside Rk1 (Zhou et al., 2020). By integrating active targets of Ginsenoside Rk1 with disease-related targets associated with inflammation and cognitive impairment, we identified 27 intersecting genes. Enrichment analyses highlighted several key inflammatory signaling cascades, including the TNF, MAPK, NF-κB, and PI3K/Akt pathways. Notably, molecular docking simulations revealed strong binding affinities between Ginsenoside Rk1 and key isoforms within the PI3K/Akt pathway (Akt1, Akt2, and Akt3). These computational insights suggested that the therapeutic effects of Ginsenoside Rk1 are predominantly mediated through the modulation of the PI3K/Akt signaling cascade, providing a rationale for our subsequent experimental validation.

To verify these predictions *in vitro*, we utilized BMDMs. Given that BMDMs and microglia share a common myeloid lineage and similar phenotypic responses to inflammatory stimuli, BMDMs serve as a robust model for studying neuroinflammatory processes (Khan et al., 2023; Ginhoux and Guiliams, 2016). Consistent with previous reports (Yu et al., 2017), our results confirmed that Ginsenoside Rk1 significantly suppressed the LPS-induced secretion of pro-inflammatory cytokines (TNF-α, IL-6, and IL-1β), establishing its potent anti-inflammatory activity at the cellular level.

Building upon these *in vitro* findings, we assessed the efficacy of Ginsenoside Rk1 *in vivo*. Based on prior dose-response studies indicating that Ginsenoside Rk1 exerts optimal efficacy at higher concentrations (Li et al., 2020; Ma et al., 2025), this high-dose regimen was selected to focus on the novel mechanistic validation of synaptic preservation rather than redundant dose-finding. MWM test revealed that LPS-challenged mice exhibited significant deficits in spatial learning and memory. Remarkably, Ginsenoside Rk1 administration effectively reversed these impairments, restoring escape latency and probe trial performance to levels comparable to controls. It is worth noting that our study design utilized a vehicle control rather than a positive drug control (such as NSAIDs). This decision was made to specifically isolate and validate the multi-target regulatory mechanisms of Ginsenoside Rk1 predicted by our network pharmacology analysis, avoiding the confounding variables introduced by standard anti-inflammatory agents that operate via distinct mechanisms (e.g., COX inhibition).

Mechanistically, we confirmed that Ginsenoside Rk1 mitigates neuroinflammation within the hippocampus, a region critical for memory consolidation. LPS-induced elevations in hippocampal pro-inflammatory cytokines and microglial activation (indicated by Iba1 staining) were significantly attenuated by Ginsenoside Rk1. This reduction in neuroinflammation is crucial, as activated microglia and inflammatory mediators are known to impair synaptic integrity (Liddelow et al., 2017).

Consequently, we observed that Ginsenoside Rk1 reversed the LPS-induced downregulation of key synaptic proteins, including PSD-95 and SYN, both critical for synaptic structure and transmission (Shen et al., 2018; Kolmogorova and Ismail, 2021; Liu et al., 2014), and restored dendritic spine density. These findings suggest that the cognitive improvement observed is likely driven by the preservation of synaptic plasticity via the suppression of neuroinflammation.

Finally, we validated the computationally predicted role of the PI3K/Akt pathway. Both in BMDMs and hippocampal tissue, LPS stimulation upregulated the mRNA expression of Akt1, Akt2, and Akt3. Ginsenoside Rk1 treatment consistently reversed these elevations. While other inflammatory pathways, such as the NLRP3 inflammasome, are undoubtedly involved in the downstream cascade of neuroinflammation, our validation focused specifically on the PI3K/Akt axis as it was identified as the primary upstream driver by our bioinformatics analysis. The consistent modulation of Akt isoforms *in vitro* and *in vivo* strongly supports the hypothesis that Ginsenoside Rk1 exerts its neuroprotective effects by regulating this specific signaling pathway.

Despite these promising findings, limitations exist. As this is an exploratory study adhering to the 4R rules (Responsibility) to minimize animal usage, future research should employ specific agonists or inhibitors of the PI3K/Akt pathway to definitively establish causality. Additionally, given the potential interactions with TNF and NF-κB pathways suggested by our docking results, further exploration of these concurrent signaling mechanisms will provide a more comprehensive understanding of the pleiotropic actions of Ginsenoside Rk1.

## 5 Conclusion

**FIGURE 7:**
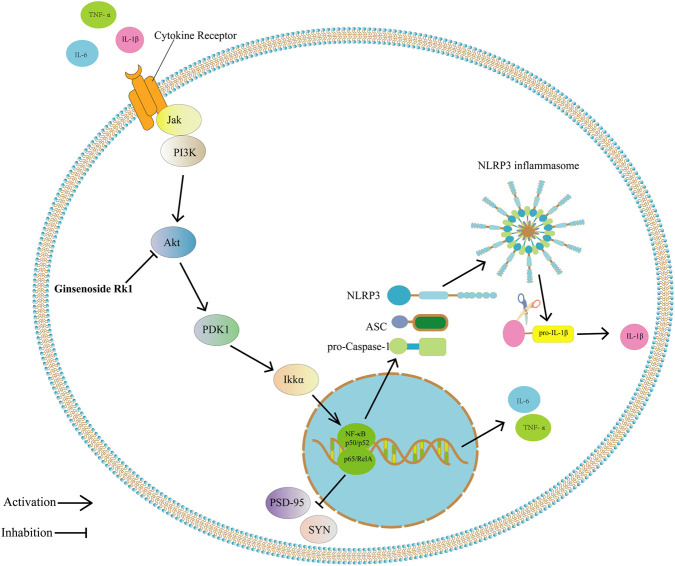
Proposed mechanism of Ginsenoside Rk1 in ameliorating LPS-induced cognitive impairment. Schematic diagram depicting how Ginsenoside Rk1 counteracts inflammation-induced cognitive dysfunction. LPS stimulation triggers the release of pro-inflammatory cytokines (IL-6, TNF-α, IL-1β) and activates intracellular signaling, specifically upregulating the PI3K/Akt pathway (Akt isoforms). This inflammatory cascade promotes NLRP3 inflammasome activation (leading to IL-1β maturation) and downregulates synaptic proteins (PSD-95, SYN), resulting in dendritic spine loss and cognitive deficits. Ginsenoside Rk1 intervention inhibits the PI3K/Akt pathway, thereby suppressing cytokine production, restoring synaptic protein expression, and preserving dendritic spine density. Arrows indicate activation/promotion; blunt lines indicate inhibition.

In conclusion, this study demonstrates that Ginsenoside Rk1 effectively alleviates LPS-induced neuroinflammation, thereby preventing associated synaptic dysfunction and ameliorating cognitive impairment (Figure 7). By integrating network pharmacology, molecular docking, and experimental validation (both *in vitro* and *in vivo*), we elucidated that Ginsenoside Rk1 exerts its neuroprotective effects, at least in part, through the modulation of the PI3K/Akt signaling pathway. These findings provide a solid theoretical and experimental foundation for the potential application of Ginsenoside Rk1 as a therapeutic agent for inflammation-related cognitive disorders.

## Data Availability

The original contributions presented in the study are included in the article/supplementary material, further inquiries can be directed to the corresponding author.
